# Age-Dependent Systemic Regulation of C1q/TNF-Related Protein 3 and Progranulin in Patients with Cystic Fibrosis: Biomarkers or Therapeutic Targets?

**DOI:** 10.3390/biomedicines14030706

**Published:** 2026-03-18

**Authors:** Andreas Schmid, Miriam Arians, Caroline Gunchick, Andreas Schäffler, Martin Roderfeld, Elke Roeb

**Affiliations:** 1 Department of Internal Medicine III, Justus Liebig University, Klinikstr. 33, 35392 Giessen, Germany; 2 Department of Gastroenterology, Justus Liebig University, Klinikstr. 33, 35392 Giessen, Germany

**Keywords:** CTRP3, progranulin, chemerin, adipokine, hepatokine, CFLD, cystic fibrosis

## Abstract

**Background/Objectives**: C1q/TNF-related protein 3 (CTRP3), progranulin (PGRN), and chemerin are adipokines that participate in systemic inflammation. This study systematically examined adipokine levels in cystic fibrosis patients of different ages to evaluate their role in inflammatory, metabolic, and hepatic processes. Thirty-seven pediatric and thirty-three adult CF patients were enrolled to assess the potential of these adipokines as biomarkers. **Methods**: Anthropometric and physiological data, pulmonary function (forced expiratory volume, FEV1; vital capacity, VC), and liver fibrosis score FIB-4 were assessed. Liver stiffness was measured by transient elastography. Serum samples from 40 healthy adult volunteers served as the control group. Serum concentrations of chemerin, CTRP3, and PGRN were quantified by enzyme-linked immunosorbent assay (ELISA). **Results**: Compared with healthy controls, adults with CF had markedly lower circulating CTRP3 levels, whereas PGRN concentrations were significantly higher. Among CF patients, both CTRP3 and PGRN were higher in the pediatric group than in adults, while chemerin did not vary with age. The presence of cystic fibrosis-related liver disease (CFLD) did not significantly alter adipokine levels relative to CF patients without liver disease. Receiver operator characteristic (ROC) analysis showed that circulating PGRN could reliably differentiate CF patients from controls; none of the three adipokines predicted the presence of CFLD. CTRP3 and PGRN were inversely correlated with age, BMI, and pulmonary function. **Conclusions**: Overall, our data support systemic PGRN as a potential biomarker for CF and indicate an age-dependent regulation of circulating CTRP3 and PGRN. Both proteins were inversely associated with BMI, inflammatory markers, liver fibrosis, and pulmonary capacity.

## 1. Introduction

Cystic fibrosis is an inherited disease that affects the exocrine glands, particularly in the gastrointestinal and respiratory tracts [1]. It leads to chronic lung disease, exocrine pancreatic insufficiency, hepatobiliary disease and pathologically high-sweat electrolytes [2]. Cystic fibrosis is the most common autosomal recessive, monogenetic disorder caused by mutations in the cystic fibrosis transmembrane conductance regulator (CFTR) gene [2]. The CFTR protein ensures that chloride ions are transported between the inner and outer space of cells [2]. However, the gene defect can disrupt this transport, which leads to dehydration of the cells and, in secretory organs, to the formation of viscous secretions like viscous mucus in the respiratory tract, digestive organs, and other loci. The lungs and pancreas are particularly affected [2]. Just a few decades ago, the life expectancy of CF patients rarely exceeded the age of twenty [3]. Today, thanks to modern treatment methods, many people with cystic fibrosis live more than twice as long [4].

Liver fibrosis is characteristic for chronic liver diseases and represents a widespread and severe health issue associated with increased premature mortality [5]. In recent decades, this pathology has mainly being caused by metabolic dysregulation such as metabolic dysfunction-associated steatotic liver disease (MASLD), especially metabolic dysfunction-associated steatohepatitis (MASH) [6], and is frequently accompanied by severe cardiovascular complications [7]. Cystic fibrosis liver disease (CFLD) is a genetically caused morbidity [8] and, in an advanced stage, comprises liver cirrhosis and portal hypertension [9]. Among individuals suffering from cystic fibrosis (CF), the prevalence of CFLD increases linearly with age [10] and represents a major cause of premature death [11,12]. In addition to the identification of serum markers in a previous proteomics approach [13], longitudinal assessment of liver stiffness recently was suggested as a non-invasive marker of progressing CFLD among juvenile CF patients [14]. According to a recent meta-analysis, liver stiffness measurement might have poor sensitivity but good specificity for detecting CFLD in CF patients [15]. Besides early-onset CFLD, CF patients are at risk of emerging and severely aggravated liver disease during adulthood [16].

While multiple interactions of the liver with adipose tissue are generally well characterized for MASLD [17], the particular role of adipose tissue-derived hormones—adipokines—in CFLD remains widely unknown. The pleiotropic adipokine C1q/TNF-related protein 3 (CTRP3) has previously been characterized as an endocrine factor involved in regulatory processes affecting multiple cell types and organs [18]. Of particular importance are the well-known anti-inflammatory properties [19,20] and the role of CTRP3 in the regulation of hepatic metabolism [21]. CTRP3 exerts beneficial effects on hepatic steatosis and triglyceride accumulation [22,23] as well as hepatic stellate cell activation [24]. Importantly, CTRP3 has previously been shown to be protective in cardiac, renal, and hepatic fibrosis [25]. This adipokine, considered an adiponectin paralog, can normalize blood glucose through several mechanisms [26]. Meanwhile, physiological data on systemic CTRP3 regulation in cystic fibrosis and associated hepatic impairment are sparse, yet crucial for a more precise characterization of its relevance in these pathologies.

Similar to CTRP3, progranulin (PGRN) is expressed in various cell types and locations including adipose tissue [27,28] and represents a multifunctional adipokine with anti-inflammatory properties [29]. A recent study by Yoo et al. demonstrated a protective role of PGRN in liver inflammation and fibrosis through inhibition of NF-κB activation [30]. Of note, progranulin has been suggested as a non-invasive marker of liver fibrosis in metabolic dysfunction-associated steatotic liver disease (MASLD), formerly known as NAFLD [31].

A further important immunomodulatory cytokine secreted by adipocytes—as well as by hepatocytes—is chemerin [32]. Mediated by its chemotactic action on immune cells [33], it is able to exert both pro- and anti-inflammatory effects, and its protective nature has been confirmed in liver disease [34].

The importance of the aforementioned adipokines in processes involved in metabolic disorders and inflammatory disease entities is still disproportional to current knowledge about their systemic regulation in cystic fibrosis and liver disease. However, an adequate understanding of these physiological and pathophysiological interactions is crucial for the identification of potential biomarkers, as well as drug targets for future CFLD therapy options. Our study aimed to gain further insight into this issue and therefore investigated the following:

Circulating CTRP3, chemerin, and PGRN levels in CF patients in comparison to a matched control group of individuals without CF or related pathologies;

The potential impact of CFLD on systemic concentrations of these adipokines among CF patients of different sex and ages;

The correlation of patients’ anthropometric characteristics with circulating adipokine concentrations.

## 2. Materials and Methods

### 2.1. Study Population

A total of 70 CF patients—36 with CFLD and 34 without liver disease including 37 pediatric (age < 18 years) and 33 adult individuals—and 40 healthy volunteers (matched by sex and BMI) were enrolled in the present study. Comprehensive data for standard anthropometric and physiological parameters were assessed for CF patients, as such data could be collected in terms of compliance and the practicability of blood sampling among pediatric/juvenile patients. Individuals with CF-associated liver disease were classified according to the extent of liver stiffness quantified by transient hepatic elastography; patients were classified as with liver disease (CFLD) or without liver disease (CFnoLD) according to the clinical guideline criteria published by Debray in 2011 as described earlier [14,35,36]. Specifically, transient elastography (TE) was performed using standardized protocols consistent with previous work by our cohort and related CF studies. A liver stiffness measurement (LSM) cut-off of approximately ≥6.8 kPa has been previously shown to distinguish CFLD from non-CFLD with good specificity and moderate sensitivity in adult CF cohorts, with an area under the curve (AUC) of ~0.87, a sensitivity of ~76% and specificity of ~92% for CFLD at this threshold. Acquisition quality was ensured by requiring a minimum number of valid TE measurements per subject in line with established elastography standards (e.g., ≥10 valid readings); acceptance of measurements only if the interquartile range (IQR)/median ratio was within generally accepted quality limits (e.g., ≤30%) to reduce technical variability; and the use of appropriate TE probes (e.g., M or XL) adapted to patient body habitus. Demographic and clinical data of the cystic fibrosis patient cohort were previously published and can be retrieved from the literature [13,14]. This study has been conducted according to the principles expressed in the Declaration of Helsinki. Written informed consent was obtained from all participating patients or their parents. The study was approved by the ethics committee of the medical faculty of the Justus-Liebig-University Giessen with the approval no. 75/09. Serum samples of 40 healthy donors were obtained from study cohorts published previously [37,38], which were approved by the ethics committee of the University Hospital Regensburg (approval no. 11-101-0058). The diagnosis of CF was established by a sweat test and later confirmed by genetic tests in all subjects. All patients were treated according to European and U.S. guidelines [39,40].

### 2.2. Enzyme-Linked Immunosorbent Assay (ELISA)

Adipokine and chemokine serum levels were quantified in technical duplicates for serum samples of CFLD patients and healthy individuals applying human-specific ELISA kits purchased from R&D Systems (Minneapolis, MN, USA). The technical detection limits and ranges were as follows:

Chemerin: 31.2–2000 pg/mL.
CTRP3: 78.1–5000 pg/mL.
MCP-1: 15.6–1000 pg/mL.
PGRN: 62.5–4000 pg/mL.
RANTES: 15.6–1000 pg/mL.

CF and control samples were collected, processed, and stored according to the same standardized protocols as the CF specimens (identical anticoagulants, processing times, centrifugation procedures, and storage at −80 °C without repeated freeze–thaw cycles). All biomarker measurements were performed in the same laboratory using identical assay platforms and reagents. In general, serum samples were thawed at room temperature prior to ELISA and, if required, dilutions were prepared from the samples under iced conditions. Dilution factors were predefined for each analyte according to pilot testing in order to ensure that measurements fell within the linear range of the assay. Samples were applied to ELISA plates in non-specific order and concentrations were determined by adding standard concentrations of the respective protein on each individual ELISA plate. Internal quality controls confirmed acceptable intra- and inter-assay variability. Measurements were repeated for all duplicates with a CV > 20%. The number of repeat measurements due to duplicate variability was low and comparable between groups.

### 2.3. Statistical Analysis

The software package SPSS 29.0 was applied for statistical data analysis. The *Mann–Whitney* U-test was applied for a comparison of metric variables in 2 unrelated samples. Graphical presentation is given as box plots (median with 25th/75th percentile) with whiskers illustrating lower and upper quartiles and dots and asterisks indicating outliers. ROC curve analysis was applied in order to identify the predictive potential of metric variables for dichotomous categories. Metric variables were analyzed by using the *Spearman-rho* rank correlation test and the respective data are presented as dot plots.

## 3. Results

### 3.1. Adult CF Patients Exhibit Lower CTRP3 and Higher PGRN Serum Concentrations than Healthy Controls

The study cohort comprised a total of 70 individuals including 37 pediatric (age < 18 years) and 33 adult (age ≥ 18 years) patients with cystic fibrosis (CF) confirmed either through a sweat test or genetic analysis. Baseline anthropometric and physiological characteristics of the study subgroup are given in Table 1. The c control group comprised 40 healthy adult individuals that were matched with the adult CF sub-cohort regarding sex and BMI (Table 1).

**Figure 1:**
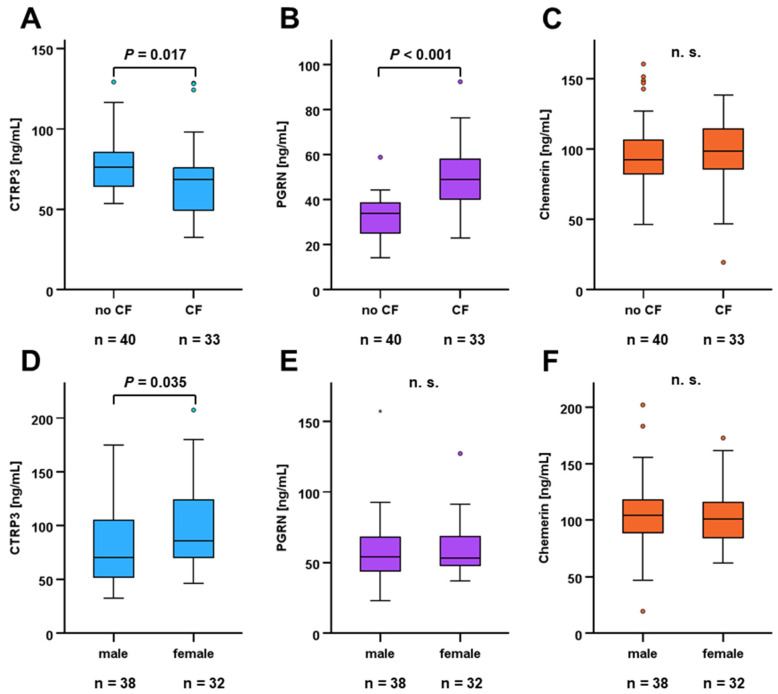
Systemic quantities of CTRP3, PGRN, and chemerin in CF patients and healthy individuals. Adipokine serum concentrations in CF patients and healthy controls were quantified via ELISA. Mann–Whitney U-test was applied in order to compare adipokine quantities for adult CF patients (n = 33) and healthy individuals (n = 40) (**A**–**C**) and for male (n = 38) and female (n = 32) CF patients (all ages included) (**D**–**F**).

Mean circulating CTRP3 levels were significantly lower in adult CF patients when compared to control individuals (68.7 ± 24.5 ng/mL vs. 78.1 ± 17.6 ng/mL; *p* = 0.017) (Figure 1A). PGRN serum concentrations were considerably elevated in CF patients (50.5 ± 13.9 ng/mL vs. 32.4 ± 9.0 ng/mL; *p* < 0.001) (Figure 1B) whereas chemerin levels were not affected (Figure 1C). Systemic CTRP3 concentrations were found to be slightly elevated in women (*p* = 0.035) (Figure 1D) whereas quantities of PGRN and chemerin did not exhibit significant sexual dimorphism (Figure 1E,F).

### 3.2. Systemic PGRN Quantities Predict Cystic Fibrosis

**Figure 2:**
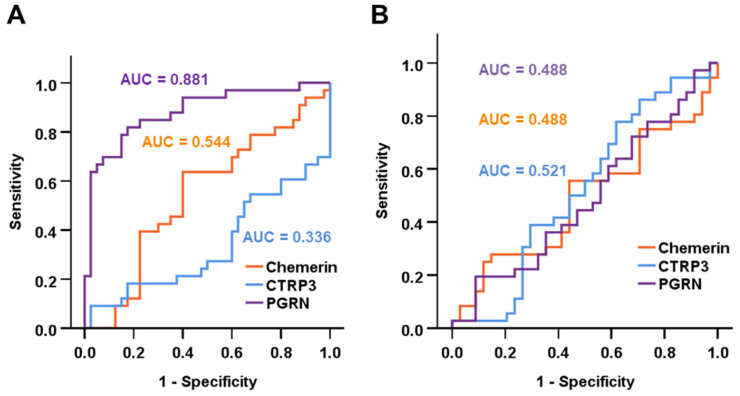
Circulating PGRN as a biomarker for CF. ROC curve analysis was applied in order to test the potential of systemic adipokine concentrations to discriminate between CF patients and healthy individuals (**A**) and to predict CFLD among CF patients (**B**).

ROC curve analysis was applied in order to identify the potential of circulating adipokine levels as biomarkers for cystic fibrosis and liver disease. Among adult subjects, PGRN serum concentrations were shown to be a valuable biomarker for discriminating between healthy individuals and CF patients (AUC = 0.884) (Figure 2A). Applying a cut-off value of 43.0 ng/mL, CF was predicted by PGRN quantities with 70.6% sensitivity and 92.5% specificity. Within the CF cohort, none of the tested adipokines was able to predict the presence of CF-associated liver disease (Figure 2B).

### 3.3. Circulating CTRP3 and PGRN Concentrations Are Elevated in Pediatric and Juvenile CF Patients

**Figure 3:**
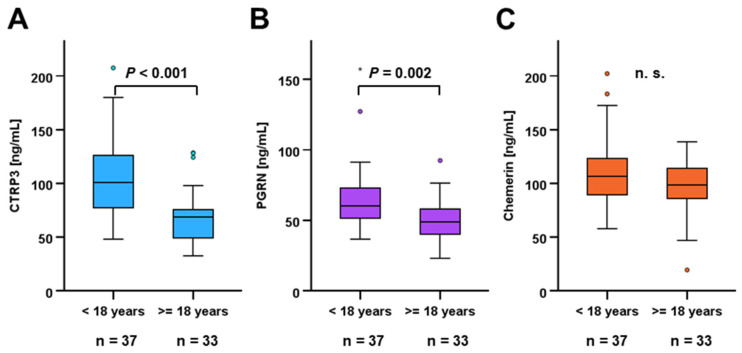
CTRP3 and PGRN serum levels are age-dependently regulated. Adipokine serum concentrations in CF patients (n = 70) were quantified via ELISA. Mann–Whitney U-test was applied in order to compare CTRP3 (**A**), PGRN (**B**), and chemerin (**C**) levels in pediatric (n = 37) and adult CF patients (n = 33).

Among CF patients, pediatric individuals exhibited substantially higher serum concentrations of CTRP3 (105.08 ± 38.61 ng/mL vs. 68.67 ± 24.50 ng/mL; *p* < 0.001) and PGRN (65.32 ± 23.02 ng/mL vs. 50.51 ± 13.90 ng/mL; *p* = 0.002) when compared to adults (Figure 3A,B). In contrast, chemerin levels did not differ between age subgroups within the entire CF cohort (111.70 ± 31.64 ng/mL vs. 97.11 ± 25.54 ng/mL; *p* = 0.150) (Figure 3C) whereas they were elevated in pediatric patients within the CFLD subgroup (*p* = 0.017).

### 3.4. Chemerin, CTRP3, and PGRN Serum Levels in CF Are Not Specifically Regulated by Liver Disease

**Figure 4:**
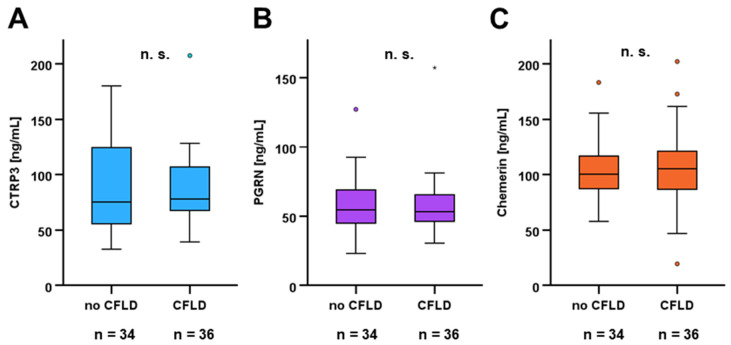
Adipokine serum concentrations in CF patients are not significantly affected by CFLD. Adipokine serum concentrations in CF patients (n = 70) were quantified via ELISA. Mann–Whitney U-test was applied in order to compare CTRP3 (**A**), PGRN (**B**), and chemerin (**C**) levels in CF patients with (n = 36) and without CFLD (n = 34) (all ages included).

Subgroup analysis was performed among pediatric and adult CF patients in order to identify potential differences between patients with and without CFLD. For the whole CF cohort (Figure 4), serum concentrations of CTRP3, PGRN, and chemerin were observed to be unaffected by additional liver disease and therefore to be non-predictive for the presence of CFLD.

In accordance with the overall findings for the total study cohort, no significant differences in adipokine concentrations between CF and CFLD patients were detected within the subgroups of pediatric and adult patients.

### 3.5. Correlations of Adipokines with Metabolic and Pathophysiological Parameters

**Figure 5:**
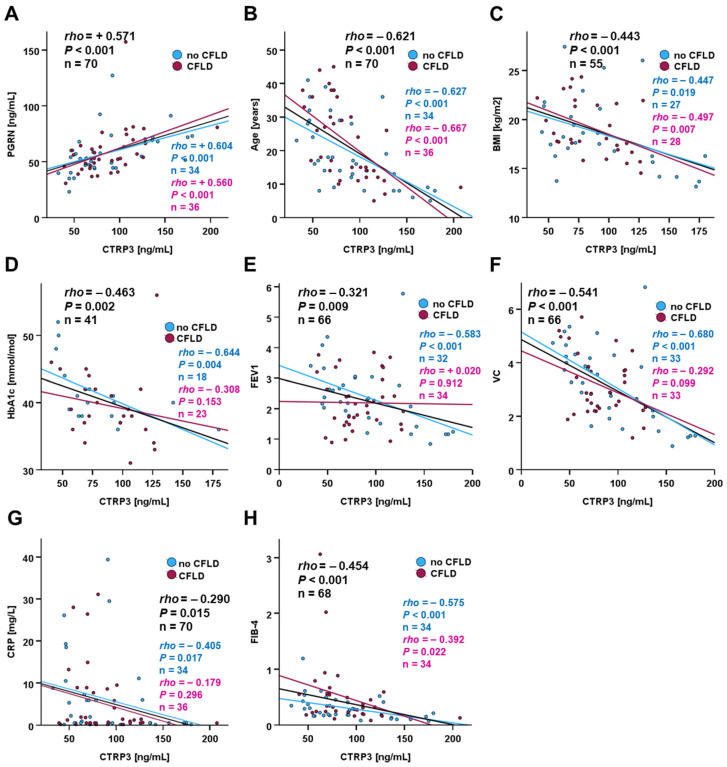
Correlation analysis of CTRP3 with anthropometric and physiological parameters: PGRN (**A**), age (**B**), BMI (**C**), HbA_1c_ (**D**), FEV (**E**), VC (**F**), CRP (**G**), FIB-4 (**H**). CTRP3 serum concentrations in CF patients (n = 70) were quantified via ELISA, and Spearman-rho test was applied in order to identify significant correlations.

Among CF patients, circulating CTRP3 and PGRN concentrations were positively correlated (rho = 0.571; *p* < 0.001) (Figure 5A) and we observed significant negative correlations of CTRP3 with age, body mass index (BMI), HbA_1c_ levels, forced expiratory volume in 1 second (FEV_1_), vital capacity (VC), C-reactive protein (CRP), and fibrosis score (FIB-4) (Figure 5B–H). While the correlations of CTRP3 with PGRN, age, BMI, and FIB-4 were not substantially affected by the presence of CFLD, the correlations with HbA_1c_, FEV_1_, VC, and CRP were found exclusively in patients without liver disease (Figure 5D–G).

**Figure 6:**
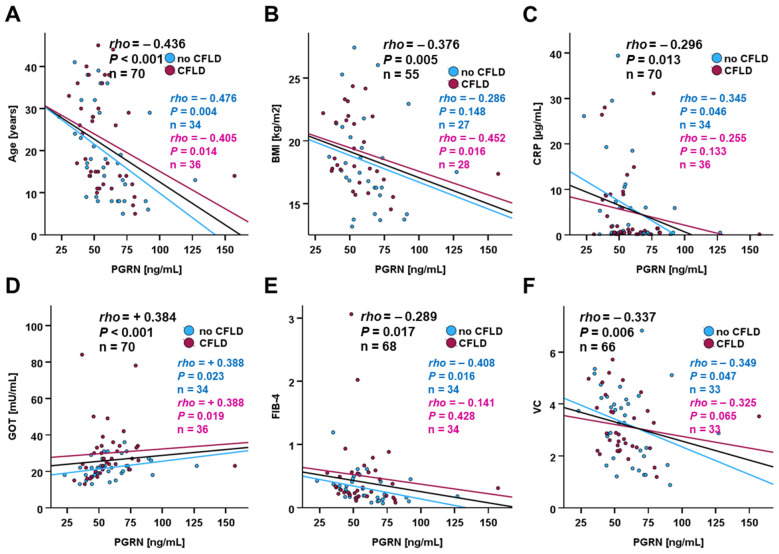
Correlation analysis of PGRN with anthropometric and physiological parameters: age (**A**), BMI (**B**), CRP (**C**), GOT (**D**), FIB-4 (**E**), VC (**F**). PGRN serum concentrations in CF patients (n = 70) were quantified via ELISA, and Spearman-rho test was applied in order to identify significant correlations.

Circulating PGRN concentrations were found to be correlated with age, BMI, CRP levels, circulating AST activity, FIB-4, and VC (Figure 6A–F). Chemerin concentrations were substantially correlated with total protein levels (rho = 0.371; *p* = 0.006), cholinesterase (rho = 0.345; *p* = 0.010), and LDH (rho = 0.445; *p* = 0.003).

Correlations of serum adipokine concentrations with anthropometric and physiological parameters for all CF patients are summarized in Table 2.

Taking into account age as a potential confounder for the observed correlations of CTRP3 and PGRN (Figure 5 and Figure 6), age-adjusted partial correlation analysis was performed for parameters of liver integrity and pulmonary function. Of note, the observed negative correlations of CTRP3 with FEV1 and VC remained significant even when adjusted for age, whereas its correlation with FIB-4, a hepatic fibrosis score, appears to be age-dependent. Furthermore, age proved to be a significant confounder for the reported negative correlations of PGRN with FIB-4 and VC.

Comparative analysis revealed that the substantial correlations of CTRP3, PGRN, and the anthropometric parameters age and BMI were absent in the control group of 40 healthy individuals.

**Figure 7:**
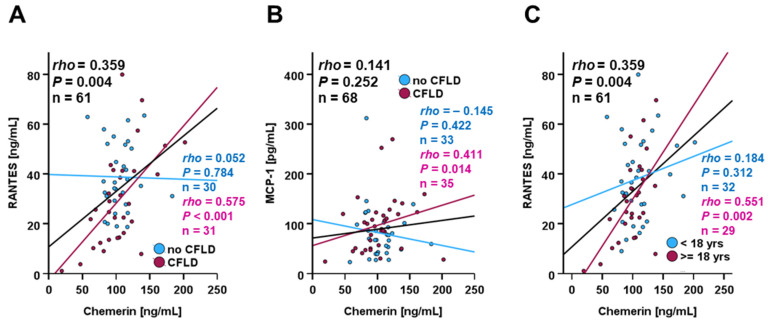
Correlation of systemic chemerin and chemokine levels. Chemerin and RANTES quantities are significantly correlated within the study cohort, depending on CFLD (**A**) and age (**C**). Chemerin and MCP-1 levels are positively correlated exclusively among CFLD patients (**B**). Chemerin, RANTES, and MCP-1 serum concentrations in CF patients (n = 70) were quantified via ELISA and Spearman-rho test was applied in order to identify significant correlations.

Systemic chemerin quantities in the entire study cohort were positively correlated with concentrations of RANTES (regulated upon activation, normal T cell expressed and secreted; chemokine (C-C motif) ligand 5, CCL5). Like chemerin, RANTES represents a chemotactic protein involved in diverse inflammatory contexts and is substantially involved in liver fibrosis [41] (rho = 0.359; *p* = 0.004) (Figure 7A). Subgroup analysis revealed that this correlation exclusively existed in CFLD patients but not in CF-only patients. Furthermore, there was also a significant positive correlation of chemerin with MCP-1 (macrophage chemoattractant protein-1; CCL2) concentrations within the CFLD sub-cohort (rho = 0.411; *p* = 0.014) (Figure 7B), i.e., with quantities of yet another chemotactic factor of particular importance in monocyte recruitment to inflamed tissues and pro-inflammatory differentiation of tissue-resident macrophages [42]. A more differentiated subgroup analysis further revealed the correlation of chemerin with CCL5 levels to be specific for adult individuals while being absent in the pediatric/juvenile sub-cohort (Figure 7C).

## 4. Discussion

Inflammatory adipokines are key players in metabolic and inflammatory diseases [43,44]. Of particular interest, adipose tissue inflammation is mechanistically linked to systemic metabolic dysregulation [45]. Detailed insight and understanding of the underlying and related processes are crucial for the development and improvement of therapeutic strategies addressing inflammatory disorders. A prior study reported altered adiponectin and resistin levels in a pediatric CF cohort [46]. The present work focused on the systemic regulation of the adipokines chemerin, CTRP3, and PGRN in pediatric and adult CF patients with or without associated liver disease (CFLD).

Our data confirm age as a general predictive factor for circulating adipokines, as has been suggested by earlier reports on correlations of age with adiponectin [47] and visfatin quantities [48]. The current study investigated age-dependent regulation of systemic CTRP3, PGRN, and chemerin concentrations in CF for the first time. CTRP3 and PGRN serum levels were observed to be substantially elevated in pediatric patients compared to adult patients, whereas chemerin quantities were not significantly associated with age within the examined CF cohort. Taking these findings into account, our data imply an age-dependent regulation of circulating CTRP3 and PGRN concentrations, starting with higher levels during childhood and adolescence and a subsequent decline during aging. The study design did not include a healthy pediatric comparator group for circulating biomarker analyses since recruitment of healthy pediatric controls was precluded due to ethical restrictions on non-therapeutic blood sampling in children. This represents an important limitation, since it remains speculative if the observed CTRP3 and PGRN concentrations are actually pathological and caused by CF or rather physiological for individuals of the respective age.

Of note, the previously described sexual dimorphism of circulating CTRP3 levels [49] was also shown by the present data, demonstrating elevated quantities in female subjects. In contrast, chemerin and PGRN serum concentrations were found to be independent of sex within the present study cohort, which is consistent with the state of knowledge retrieved from the current literature.

When compared to healthy individuals matched for sex and BMI, the three adipokines exhibited very different regulatory patterns in CF. While circulating CTRP3 concentrations were observed to be markedly decreased in CF patients, PGRN levels were elevated and chemerin quantities were not affected. These findings argue for a divergent regulation of CTRP3, chemerin, and PGRN in CF.

CTRP3 is considered an adipokine with well-known anti-inflammatory characteristics in diverse pathological contexts and mechanisms [18,20]. While previous studies reported an inverse proportionality of circulating CTRP3 and fatty liver disease [50,51] as well as hepatic fibrosis [25], there is a lack of data on its potential involvement in cystic fibrosis and related liver disease. Our current data show reduced CTRP3 levels in adult CF patients when compared to healthy individuals. Of note, no significant difference was observed among CF patients when comparing subjects with and without liver disease. This finding is rather surprising regarding the downregulation of CTRP3 in fibrotic livers observed in a previous study [25] and implies that additional hepatic impairment does not further enhance the observed CTRP3-decreasing effect of CF per se. Given previously reported anti-fibrotic effects of CTRP3 [25,52,53], reduced serum quantities in the overall CF cohort might indicate a more general predisposition to conditions potentially promoting fibrosis. However, the molecular mechanisms underlying the putative effects of fibrosis remain unclear and have to be addressed by subsequent research elaborating on the present data. Since the healthy individuals being studied as a control group were matched with the cohort of adult CF patients for sex and BMI, these parameters can be excluded as potential confounders affecting the observed divergence in CTRP3 quantities. Since there was a moderate yet significant difference in mean age between both groups (CF cohort: 31.45 years; control group: 26.98 years) (Table 1), a contribution of age-dependent effects to higher CTRP3 concentrations in the healthy subjects cannot completely be ruled out due to the aforementioned association of CTRP3 levels with age. However, since no significant correlation between CTRP3 and age was found within the control group, and regarding the rather small age gap, we do not assume age to be a substantial cause for the higher CTRP3 quantities observed in the CF cohort. Nevertheless, effects associated with differing mean age might contribute to the observed difference in adipokine quantities, which should therefore be validated in future studies comparing CF cohorts with precisely age-matched controls. Regarding its general anti-inflammatory characteristics, it appears reasonable to speculate on a protective role of CTRP3 in cystic fibrosis. Yet again, further experimental approaches on molecular and cellular levels are required in order to elucidate CTRP3 as a distinguished anti-fibrotic target in CF. Furthermore, future clinical studies in larger CF cohorts should address correlations of CTRP3 levels with pro-inflammatory cytokines such as interleukin 6 (IL6) and tumor necrosis factor (TNF) as well as potential effects of liver disease on this putative association. Of note, there was no significant predictive potential of CTRP3 for the discrimination of CF patients from healthy individuals, thus arguing against this adipokine as a valid biomarker for the detection and validation of CF.

PGRN has previously been reported as a biomarker being elevated in MASLD [31] and exerting anti-inflammatory effects in liver fibrosis [30]. On the other hand, progranulin contributes to insulin resistance and the resulting deterioration of carbohydrate metabolism [27]. Thus, progranulin has both detrimental and beneficial effects. The observed upregulation among CF patients might be associated with inflammation, potentially as a part of protective mechanisms. Based on the observed association of elevated systemic PGRN quantities with CF, ROC analysis revealed its potential as a valuable biomarker. A PGRN cut-off value of 43.0 ng/mL was found to predict the presence of CF with high specificity and acceptable sensitivity. Of note, due to its more general involvement in inflammation [54], further clinical research is needed to clarify whether PGRN can be considered a novel biomarker specific for CF. Future studies elaborating on the current findings should verify its applicability with the suggested cut-off level in larger clinical cohorts.

A finding of particular interest is that the additional CFLD diagnosis was not associated with significant differences in all three adipokines’ serum levels when compared to patients solely suffering from CF. Therefore, the presence of additional liver disease does not appear to further exacerbate the effects on systemic CTRP3 and PGRN levels caused by lung damage in cystic fibrosis alone. This finding might argue for fibrosis-related processes being crucial for CTRP3 downregulation and PGRN upregulation rather than impaired hepatic processes. Again, the present data should motivate appropriate study designs in order to evaluate this hypothesis and elucidate underlying mechanisms.

A positive correlation of CTRP3 and PGRN serum levels was detected within the total CF cohort and suggests putative co-regulation of both adipokines. These data are in accordance with our previous findings in a study on severely obese adult individuals [55]. Negative correlations with age further support the hypothesis of elevated CTRP3 and PGRN quantities at an earlier age.

The observed negative correlations of CTRP3 with BMI, HbA_1c_ levels, and FIB-4, a score for liver fibrosis, are in accordance with the established association of this adipokine with parameters of metabolic health and liver integrity [21,25,56]. Of note, the correlation between CTRP3 and FIB-4 depended on age as a significant confounding factor. Taken together, it appears reasonable to consider reduced circulating CTRP3 quantities as a biomarker indicating a rather impaired and dysbalanced overall metabolic phenotype. Similarly, negative correlations of PGRN concentrations with BMI and CRP levels suggest an association with immune–metabolic balance. Of particular interest, elevated PGRN concentrations were found in individuals with a reduced risk of liver fibrosis and dysfunction. This observation is in good accordance with the protective PGRN effects reported previously [30]. Older studies, however, described elevated PGRN levels as a fibrosis marker in NAFLD patients [31], and the implications for the overall role of PGRN in liver injury still remain somewhat controversial. Serum PGRN concentrations were negatively correlated with FIB-4, indicating an association of elevated circulating levels with a lower risk of liver fibrosis. At first glance, this observation is rather surprising since systemic PGRN has previously been reported to be a biomarker for the progression of liver fibrosis [31]. These apparently contradictory findings might be explained by the divergent disease context of NAFLD as a metabolic disorder and of CF-related liver fibrosis in the present study cohort. Future research involving comparative clinical studies should therefore address and clarify the issue of PGRN regulation in different etiologies of liver fibrosis.

Of note, a recent study pointed out the essential role of PGRN in macrophage efferocytosis through regulation of cystic fibrosis transmembrane conductance regulator (CFTR)-dependent lysosomal acidification [57]. Thus, the increased systemic PGRN quantities that we observed in CF patients might be causally linked to deficient macrophage activity in CF progression [58] as a potential compensatory mechanism. However, an association with increased fibrosis and inflammation remains somewhat speculative due to the lack of a substantial correlation of PGRN with pro-inflammatory parameters such as CRP and MCP-1.

Contrary to the observations regarding CTRP3 and PGRN, chemerin did not significantly correlate with metabolic parameters, whereas substantial correlations with the chemotactic factors MCP-1 and RANTES were observed among CFLD patients. The latter might indicate the involvement of chemerin in inflammatory processes such as immune cell recruitment and activation, especially in CFLD, presumably affecting liver function and integrity in respective patients.

Whereas circulating chemerin is known to be linked to MASLD [59], the present data do not indicate an association of chemerin quantities with liver disease among CF patients. Our observations are consistent with those previously anticipated in another cohort [60]. Chemerin, an adipokine primarily secreted by hepatocytes and adipose tissue, modulates hepatic lipid metabolism and insulin sensitivity while also acting as a chemotactic signal for immune cells, thereby linking metabolic regulation with local inflammatory responses in the liver. Chemerin does not appear to serve as a general marker of systemic inflammation, but rather reflects metabolic dysregulation and adipose tissue-associated inflammatory processes. Consequently, circulating chemerin levels remain within the normal range in patients with cystic fibrosis, despite chronic pulmonary inflammation. Future studies enrolling larger numbers of CF/CFLD patients will be needed in order to more validly address this important issue.

Of particular interest, we observed negative correlations of FEV1 with CTRP3 and of VC with CTRP3 and PGRN levels, indicating a potential association of both adipokines with impaired pulmonary performance. Of note, the adipokines adiponectin and CTRP5, which are paralogous to CTRP3, have previously been reported to be associated with the severity of chronic obstructive pulmonary disease (COPD) [61,62]. But no such association was found for CTRP3 [62]. Thus, our current data might indicate a more general correlation of CTRPs with impaired lung function. In order to further elucidate this intriguing issue and to evaluate the potential role of CTRP3 and PGRN as biomarkers for pulmonary disease, prospective clinical studies in adequate cohorts of pulmonary diseases are needed.

A limitation of the present study refers to the applied method of liver stiffness measurement. While LSM correlates with the presence and severity of CFLD, its sensitivity for early or mild disease is limited and varies across studies. Consequently, non-differential misclassification of mild CFLD using LSM is possible, tending to bias subgroup comparisons toward the null hypothesis. Thus, the reported findings of no significant differences by CFLD status should be interpreted with caution and in the context of the aforementioned limitations of TE sensitivity for early/less advanced CFLD. Complementary diagnostic approaches—such as longitudinal LSM changes and biochemical indices—might enhance disease detection in future studies.

Examining a set of adipokines and physiological parameters, analysis of the present data includes numerous comparison tests and correlation analyses. Importantly, the multiplicity of statistical tests might affect the validity of the reported results. While highly significant inter-group differences and bivariate correlations (*p* < 0.001) are considered valid results, those with a lower level of significance should be interpreted with caution regarding their biological and clinical relevance. In general, the findings of the current study are presented as a conceptual perspective that warrants further experimental validation, rather than as a definitive conclusion. In general, we provide the results of exploratory analyses, potentially affected by confounding parameters, particularly age. Therefore, the reported data should motivate future confirmatory studies employing formal multivariable adjustment and pre-specified endpoints. Similarly, the presented ROC analysis was conducted within an a priori clinically classified cohort. The suggested cut-off in PGRN concentrations should be interpreted as illustrative and hypothesis-generating rather than as a validated clinical decision point, requiring further elaboration and validation by upcoming studies in larger CF cohorts and control groups.

## 5. Conclusions

In adult individuals, CF is associated with lower CTRP3 and elevated PGRN serum levels whereas chemerin quantities are not affected. Among CF patients, the presence of CF-associated liver disease (CFLD) does not additionally affect the systemic regulation of these immunomodulatory adipokines. Within CF, age appears to be a significant factor of CTRP3 and PGRN regulation, as indicated by elevated circulating concentrations of both adipokines in pediatric and juvenile patients when compared to adults. CTRP3 and PGRN might be involved in—or regulated by—impaired pulmonary function in CF. In conclusion, we are considering PGRN a novel CF biomarker especially for younger patients.

## Figures and Tables

**Table 1:** Standard anthropometric and physiological data of pediatric (A) and adult (B) CF patients and of adult controls (C). Mean values ± standard deviation are listed. ALT, alanine aminotransferase; AST, aspartate aminotransferase; BMI, body mass index; HbA_1c_, hemoglobin A_1c_; GGT, gamma-glutamyl transferase; * *p* < 0.05 vs. B.

	A (n = 37)	B (n = 33)	C (n = 40)
Age [years]	11.95 ± 3.70	31.45 ± 7.19	26.98 ± 3.04 *
Sex	18 male (48.6%)	20 male (60.6%)	24 male (60%)
	19 female (51.4%)	13 female (39.4%)	16 female (40%)
Body weight [kg]	38.33 ± 13.73	62.33 ± 11.50	
BMI [kg/m^2^]	17.46 ± 2.94	21.05 ± 2.76	21.70 ± 2.53
HbA_1c_ [mmol/mol]	38.76 ± 3.36	42.87 ± 6.24	
ALT [U/L]	32.62 ± 33.04	26.94 ± 10.44	
AST [U/L]	28.57 ± 14.56	23.45 ± 8.64	
GGT [U/L]	18.00 ± 18.71	24.63 ± 23.24	

**Table 2:** Correlations of CTRP3, PGRN, and chemerin with anthropometric and physiological parameters. Non-parametric Spearman-rho test was applied for calculation of correlation coefficients and statistical significance.

	CTRP3		PGRN		Chemerin	
	rho	*p*	rho	*p*	rho	*p*
Age	−0.621	<0.001	−0.436	<0.001	−0.174	0.149
BMI	−0.443	<0.001	−0.376	0.005	+0.019	0.893
Protein	−0.075	0.591	−0.135	0.332	+0.371	0.006
Albumin	+0.478	<0.001	+0.215	0.134	+0.216	0.133
AST	+0.265	0.027	+0.384	0.001	+0.240	0.046
FIB-4	−0.454	<0.001	−0.289	0.017	−0.148	0.229
Cholinesterase	+0.291	0.031	+0.299	0.027	+0.345	0.010
AP	+0.549	<0.001	+0.515	<0.001	+0.221	0.159
CRP	−0.290	0.015	−0.296	0.013	+0.223	0.064
LDH	+0.228	0.141	+0.180	0.247	+0.445	0.003

## Data Availability

The data presented in this study are available on request from the corresponding author.
